# Integrative analysis of genomic amplification-dependent expression and loss-of-function screen identifies ASAP1 as a driver gene in triple-negative breast cancer progression

**DOI:** 10.1038/s41388-020-1279-3

**Published:** 2020-03-31

**Authors:** Jichao He, Ronan P. McLaughlin, Lambert van der Beek, Sander Canisius, Lodewyk Wessels, Marcel Smid, John W. M. Martens, John A. Foekens, Yinghui Zhang, Bob van de Water

**Affiliations:** 1 Division of Drug Discovery and Safety, Leiden Academic Centre for Drug Research, Leiden University, 2300 RA Leiden, The Netherlands; 2 Division of Molecular Carcinogenesis, Netherlands Cancer Institute, 1066 CX Amsterdam, The Netherlands; 3 Department of Medical Oncology and Cancer Genomic Netherlands, Erasmus MC Cancer Institute, Erasmus Medical Centre, 3000 CA Rotterdam, The Netherlands

**Keywords:** Cancer genomics, Breast cancer

## Abstract

The genetically heterogeneous triple-negative breast cancer (TNBC) continues to be an intractable disease, due to lack of effective targeted therapies. Gene amplification is a major event in tumorigenesis. Genes with amplification-dependent expression are being explored as therapeutic targets for cancer treatment. In this study, we have applied Analytical Multi-scale Identification of Recurring Events analysis and transcript quantification in the TNBC genome across 222 TNBC tumors and identified 138 candidate genes with positive correlation in copy number gain (CNG) and gene expression. siRNA-based loss-of-function screen of the candidate genes has validated EGFR, MYC, ASAP1, IRF2BP2, and CCT5 genes as drivers promoting proliferation in different TNBC cells. MYC, ASAP1, IRF2BP2, and CCT5 display frequent CNG and concurrent expression over 2173 breast cancer tumors (cBioPortal dataset). More frequently are MYC and ASAP1 amplified in TNBC tumors (>30%, *n* = 320). In particular, high expression of ASAP1, the ADP-ribosylation factor GTPase-activating protein, is significantly related to poor metastatic relapse-free survival of TNBC patients (*n* = 257, bc-GenExMiner). Furthermore, we have revealed that silencing of ASAP1 modulates numerous cytokine and apoptosis signaling components, such as IL1B, TRAF1, AIFM2, and MAP3K11 that are clinically relevant to survival outcomes of TNBC patients. ASAP1 has been reported to promote invasion and metastasis in various cancer cells. Our findings that ASAP1 is an amplification-dependent TNBC driver gene promoting TNBC cell proliferation, functioning upstream apoptosis components, and correlating to clinical outcomes of TNBC patients, support ASAP1 as a potential actionable target for TNBC treatment.

## Introduction

Triple-negative breast cancer (TNBC) represents a particularly proliferative and aggressive subtype of breast cancer (BC), associated with large size of tumors, high mitotic rate of tumor cells, high tumor grade and metastasis, causing poor prognosis and high mortality rate of patients [1]. TNBC constitutes 15–20% of BC, being clinically characterized by the lack of expression of estrogen receptor (ER), progesterone receptor (PR), and the absence of amplification of human epidermal growth factor receptor 2 (HER2), which are the known drivers of other BC types [2]. The absence of actionable targets defined in TNBC cells leads to clinical failure of targeted therapies in TNBC patients. Cytotoxic chemotherapy remains the conventional systemic treatment for TNBC patients, resulting in adverse effects and unfavorable outcomes [3, 4]. Identification of actionable targets for TNBC treatment is a continuous effort.

Gene copy number alterations (CNAs) are a hallmark in the cancer genome [5]. Gains or losses of gene copy are important somatic genomic aberrations contributing to tumorigenesis [6]. Changes in gene copy number result in corresponding changes in expression of the affected genes, causing phenotypes [7]. Copy number gains (CNGs), increasing from the two DNA copies present in normal diploid genome, sometimes to several hundred copies (known as amplification), are observed to frequently occur on cancer driver genes [8, 9]. Oncogenic driver genes with increase in DNA copy number and expression have been identified and explored as potential drug targets for targeted therapies in cancer [10]. For instance, the *HER2* gene, which is amplified in ~30% of primary BCs [11], has been proven as an actionable target for trastuzumab antibody targeted therapy and lapatinib inhibitor targeted therapy treating patients with HER2-amplified BC [12]. Therefore, identification of CNG-driven genes and their amplification-dependent overexpression provides opportunities for discovering potential cancer driver genes as therapeutic targets for therapy-refractory cancer.

A number of studies have demonstrated the genomic heterogeneity in TNBCs, being dominated by substantial mutational burdens, including CNAs and genomic rearrangements [6, 13]. CNA genomic profiling based on separate TNBC sample groups have reported the TNBC-related recurrent CNAs on various chromosome regions [14]. Yet, integrative analysis of CNG frequency and CNG-driven gene expression in the TNBC genome is limited.

In this study, we applied the Analytical Multi-scale Identification of Recurring Events (ADMIRE) algorithm to identify candidate driver genes that are frequently amplified by recurrent CNGs, in correlation with their RNA expression levels, in 222 triple-negative tumors from TCGA (*n* = 118) [15] and Metabric (*n* = 104) [2] datasets. As a result, 138 genes were identified, with a significant and positive correlation in their gene amplification and expression. These amplification-driven genes were subsequently validated by loss-of-function screen for their biological function in TNBC cell proliferation, followed by assessment of their expression and amplification in broad BC cell lines and tumors, and evaluation of their clinical relevance using multiple large public BC datasets [16, 17]. Consequently, we characterized the known oncogenes MYC and EGFR and the novel candidate genes ASAP1, IRF2BP2, and CCT5 as cancer drivers in promoting proliferation of TNBC cells. MYC and ASAP1 were observed to be more frequently amplified and highly expressed in TNBC than non-TNBC tumors. Specifically, high expression of ASAP1, an ADP-ribosylation factor (Arf) GTPase-activation protein regulating cell motility and invasiveness [18], is significantly relevant to poor metastatic relapse-free survival (MRFS) of patients with TNBC tumors, not non-TNBC tumors. Transcriptome analysis further revealed that ASAP1 regulates various cytokine and apoptosis signaling components that are significantly associated with TNBC prognosis. Our work discovered ASAP1 as an amplification-dependent gene driving TNBC proliferation, survival and progression, supporting the potentiality of ASAP1 as a therapeutic target for the treatment of TNBC.

## Results

### ADMIRE analysis of TNBC genomes identifies TNBC candidate driver genes with recurrent copy number gain and correlated expression

**Fig. 1:**
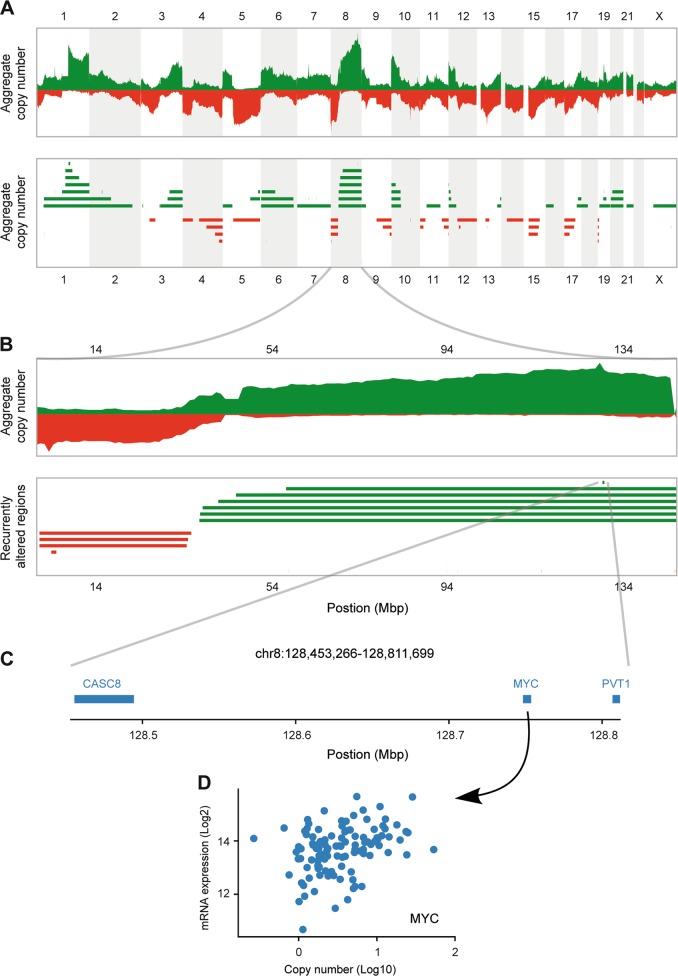
Schematic selection of candidate driver genes with increases in copy number and expression in TNBC genome. **a** Discovery of genomic regions with recurrent copy number alterations using ADMIRE analysis. The top panel shows the aggregate copy number profile across 222 triple-negative breast tumors (*n* = 118 for TCGA; *n* = 104 for Metabric). The bottom panel shows the significant recurrent copy number regions, with gains in green and losses in red. **b** Zoomed in fragment of panel **a**, focusing on chromosome 8. The bottom panel reveals the small focal recurrent copy number gain on 8q for further analysis. **c** The genes contained in the focal region identified in panel **b**. **d** Scatterplot exemplifying the positive correlation of MYC gene expression with its copy number in TNBC patients.

Recurrent CNAs have been recognized as the result of natural selection in tumor evolution, and hence the recurrently altered regions are likely to harbor cancer driver genes [19]. In order to identify candidate driver genes for TNBC, we applied ADMIRE, a robust algorithm for the discovery of broad and focal recurring events [20], to detect genomic regions with frequent CNAs in a set of TNBC tumors (*n* = 118 for TCGA; *n* = 104 for Metabric). Aggregated DNA copy number profiles (Fig. 1a) assisted in pinpointing recurrently altered regions (Fig. 1b). Genes contained in the focal regions were further assessed with copy number and expression correlation analysis (Fig. 1c). Genes showing positive correlation in mRNA expression levels and copy numbers were filtered out as candidate drivers, as exemplified for the proto-oncogene MYC (Fig. 1d). The ADMIRE analysis and the subsequent filtering steps were performed separately for TCGA and Metabric cohorts. Subsequently, 138 genes were selected as candidate driver genes for TNBC (Supplementary Table 1). Next, functional enrichment analysis displayed the significant implication of the 138 genes in 18 KEGG pathways (Supplementary Fig. 1). The 138 gene set was enriched in cancer-related pathways, including central carbon metabolism in cancer, proteoglycans in cancer, pathways in cancer, melanoma, prostate cancer, and endometrial cancer. Moreover, these candidate drivers were implicated in various oncogenic signaling pathways that control cell growth, survival, and motility, such as ErbB, PI3K-Akt, Ras, MAPK, and Wnt pathways. Altogether, our ADMIRE genomics approach collected 138 candidate TNBC drivers that are frequently amplified by recurrent CNGs in the TNBC genome and significantly enriched in pathways that fuel cancer progression.

### Loss-of-function screen validates the TNBC candidate driver genes in TNBC cell proliferation

**Fig. 2:**
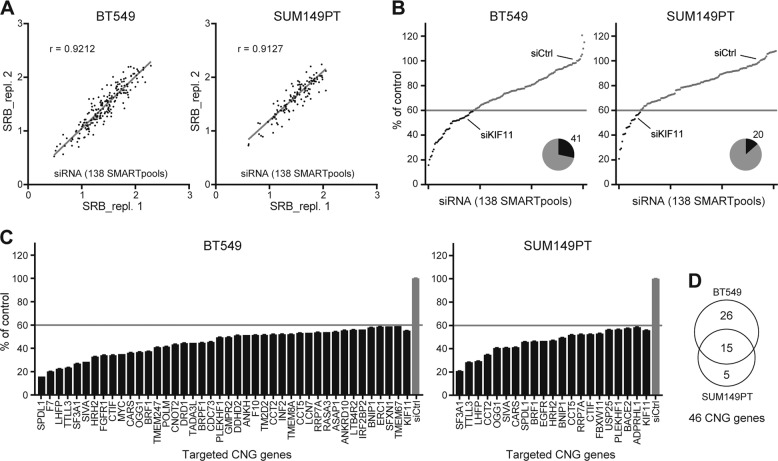
siRNA-mediated loss-of-function screen of candidate driver genes in TNBC cells. **a** Replicate siRNA screens of candidate driver genes in TNBC cell lines BT549 and SUM149T. siRNA silencing effect of candidate gene on cell proliferation was assessed 96 h after transfection and presented with sulforhodamine B (SRB) colorimetric raw values. **b** Normalized percentage of proliferation control by siRNA silencing. The number of genes (black) whose silencing led to >40% proliferative inhibition was indicated in the pie chart, as primary hits. **c** Ranking and listing of the primary hits significantly controlling proliferation of BT549 and SUM149PT TNBC cell lines. siRNA targeting KIF11, positive functional control; siCtrl, non-targeting siRNA control. siRNA silencing effect on proliferation was relative to siCtrl. Error bars indicate variation of screen replicates. **d** Overlap primary hits in BT549 and SUM149T TNBC cell lines.

As a first step, we assessed the biological function of the 138 candidate genes in control of TNBC cell proliferation by siRNA-mediated loss-of-function screen in two TNBC cell lines, mesenchymal-like BT549 and basal-like SUM149T. High reproducibility was achieved for the duplicate screens in BT549 (*r* = 0.9212) and SUM149PT (*r* = 0.9127) (Fig. 2a). Genes whose silencing controlled proliferation <60% were considered as candidate hits with significance (Fig. 2b). Consequently, 41 primary hits were screened for BT549 and 20 for SUM149PT cell line (Fig. 2b, c). In total, 46 primary hits were selected, of which 15 were common (Fig. 2d; Supplementary Table 2).

**Fig. 3:**
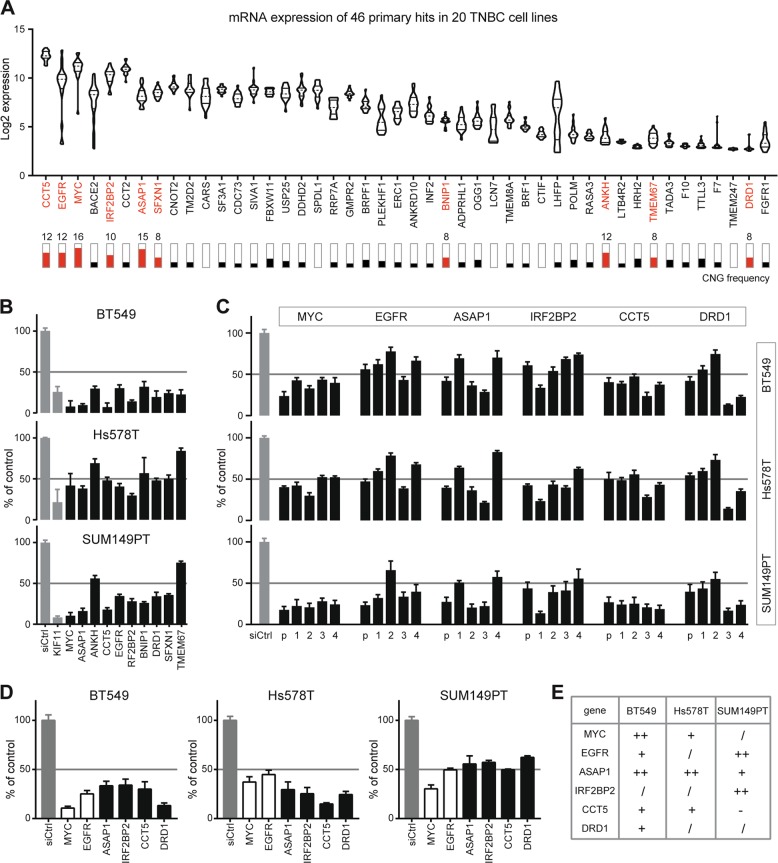
Validation of candidate driver genes with concurrent copy number gain (CNG) and overexpression in TNBC cells. **a** mRNA expression of 46 primary hits in 20 TNBC cell lines. Violin plot indicates Log2 mRNA expression level of 46 primary hits in 20 TNBC cell lines retrieved from RNA-Seq analysis. Bars indicate CNG frequency of the hits in 20 TNBC cell lines. Genes with frequent CNG in ≥8/20 TNBC cell lines were marked in red. **b** siRNA validation of primary candidate hits with high frequent CNG in BT549, Hs578T, and SUM149PT TNBC cell lines. SMARTpool siRNAs were used to target each hit. KIF11 was taken as positive control. **c** siRNA deconvolution validation of six candidate driver hits. The effects of SMARTpool (p) siRNA and single siRNA_1, _2, _3, and _4 on hits were compared for their proliferation control (%) in the TNBC cell lines. **d** Percentage of control proliferation (%) by optimized SMARTpool siRNAs targeting the six candidate hits. The results were expressed as mean ± SEM of three independent experiments. **e** CNA of the six candidate driver genes in TNBC cell panel. “++”, high CNG; “+”, CNG; “/”, no copy number alteration; “−”, copy number loss.

Next, we examined the CNG frequency of the 46 primary hits in 20 TNBC cell lines, representative for diverse molecular subtypes of TNBC. Ten candidate hits were found with CNG frequency across ≥8 TNBC cell lines (Fig. 3a, lower panel). The CNG recurrence for MYC was found in 16/20 of TNBC cell lines, ASAP1 in 15/20, ANKH, CCT5, and EGFR in 12/20, IRF2BP2 in 10/20, BNIP1, DRD1, SFXN1, and TMEM67 in 8/20, respectively. We also evaluated the mRNA expression level of the 46 primary hits in the 20 TNBC cell line panel in our established RNA-Seq data (Fig. 3a, upper panel). Given the nature of heterogeneity of TNBC, the correlation of expression and copy number of the primary hits varied among cell lines.

To further validate the ten candidate hits for their function in proliferation, we performed siRNA silencing in three TNBC cell lines, BT549, SUM149T, and one more mesenchymal-like cell line Hs578T. Silencing of six hits, ASAP1, CCT5, IRF2BP2, DRD1, and two known oncogenes MYC and EGFR, potently inhibited proliferation (>50%) in all three cell lines (Fig. 3b), suggesting their driving role in TNBC cell proliferation. Deconvolution siRNA screen confirmed the effect of single siRNAs (≥2/4) on the proliferation-driving hits, mostly achieving >50% of proliferation inhibition, ruling out the off-targeting effect of pooled siRNAs (Fig. 3c). The optimized pooled siRNA silencing further validated the function of the driver hits in proliferation control (>50%) (Fig. 3d). Of note, the inhibitory effects on cell proliferation by silencing these six hits were, in general, concordant with CNA status in these cell lines (Fig. 3e).

Collectively, our RNAi-based functional screen validated the ADMIRE-identified candidate driver genes and defined the role of MYC, EGFR, ASAP1, IRF2BP2, CCT5, and DRD1 in controlling proliferation of TNBC cells.

### Frequent amplification of ASAP1 in TNBC is significantly relevant to poor clinical outcome

**Fig. 4:**
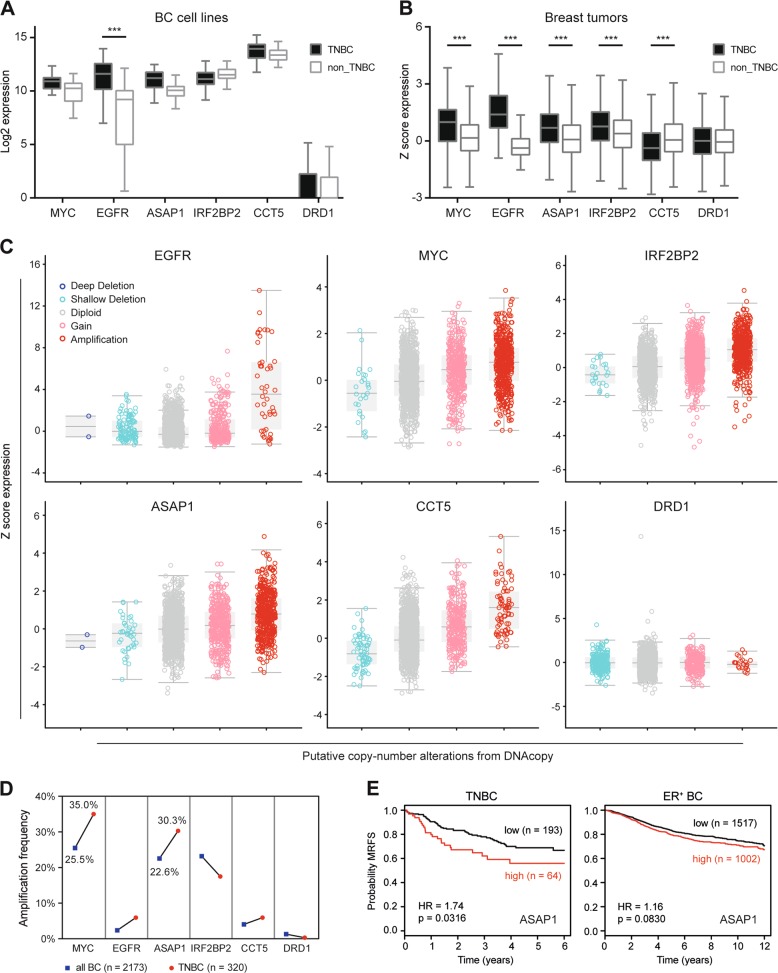
ASAP1 amplification and overexpression in TNBC in association with poor clinical outcome. **a** Log2-based mRNA expression of the six candidate hits in 52 breast cancer (BC) cell lines. Log2 values were obtained from established RNA-Seq data (two-way ANOVA ***p < 0.001). **b** *Z* score-based mRNA expression of the hits in 1904 BC tumors. Data were retrieved from the dataset “METABRIC, Nature 2012 & Nat Commun 2016” in cBioPortal dataset. **c** Correlation between CNA and gene expression of the candidate hits in the cohort of 1904 BC tumors. Different copy number amplifications (deep deletion, shallow deletion, diploid, gain, and amplification) are presented per gene. **d** Amplification frequency of the candidate hits in the cohort of 2173 BC tumors. Data were retrieved from the dataset “METABRIC, Nature 2012 & Nat Commun 2016” in cBioPortal dataset. **e** MRFS Kaplan–Meier (KM) curve of ASAP1 in TNBC (*n* = 257) and ER^+^ BC (*n* = 2519) cohorts analyzed by bc-GenExMiner v4.2.

We next sought to address the correlation between CNA frequencies and expression levels of MYC, EGFR, ASAP1, IRF2BP2, CCT5, and DRD1 in broad BC cell lines and breast tumors and the clinical relevance of the genes to patients with BC. RNA-Seq analyses of the transcriptome from our 52 BC cell lines demonstrated that the Log2-based mRNA expression levels of MYC, EGFR, ASAP1, and CCT5 were higher in TNBC than non-TNBC cell lines (Supplementary Table 3), while similar high IRF2BP2 and low DRD1 were expressed in both cell types (Fig. 4a). The difference of EGFR expression in TNBC and non-TNBC cells was of significance. Next, we obtained *Z* score-based mRNA expression data for the hits in 1904 BC tumors from cBioPortal database [2, 17, 21]. MYC, EGFR, ASAP1 and IRF2BP2 were highly expressed in TNBC, compared with non-TNBC tumors (Fig. 4b). CCT5 expression was found lower in TNBC than non-TNBC breast tumors, and DRD1 showed no significant difference. We further illuminated the correlation between CNAs and expression levels of the genes in the cohort of 1904 BC tumors. While CNA events in deep or shallow depletion rarely or less occurred, EGFR, MYC, IRF2BP2, ASAP1, and CCT5 (except for DRD1) often underwent copy gain and amplification, acquiring CNA-driven expression in the breast tumors (Fig. 4c). Moreover, MYC, ASAP1, and IRF2BP2 were amplified in >20% of all 2173 BC tumors (Fig. 4d). Particularly, MYC and ASAP1 amplifications emerged more frequently in TNBC tumors (>30%), suggesting TNBC subtype-related MYC and ASAP1 amplifications. Prognostic analysis demonstrated the association of the CNA-driven hits with overall survival (OS) of the cohort of 1981 BC patients [2, 21], indicating the significant implication of MYC and ASAP1 in poor disease outcomes (Supplementary Fig. 2). More specifically, high ASAP1 expression was revealed to be related to worse MRFS of patients with TNBC (*n* = 257), but not ER^+^ BC (*n* = 2519) (Fig. 4e), as assessed in the cohorts of BC patients [16]. Together, we characterized MYC and ASAP1 as CNA-driven genes with frequent amplification and concurrent high expression in BC tumors. The CNA-driven amplification and expression of ASAP1 exhibited significant clinical impact particularly on TNBC progression.

### Transcriptomic analysis of the impact of ASAP1 depletion on gene expression in TNBC cells

**Fig. 5:**
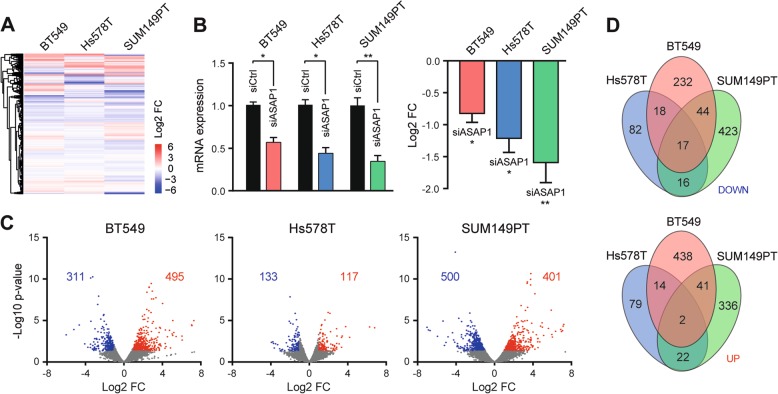
Targeted whole transcriptome analysis of ASAP1 depletion-induced transcription reprogramming in TNBC cells. **a** Transcriptome expression profiling in BT549, Hs578T and SUM149PT TNBC cells after siRNA-mediated depletion of ASAP1 (siASAP1). Log2 fold change (Log2 FC), siASAP1 versus siCtrl. **b** Targeting effect of siASAP1 on ASAP1 gene expression. Left panel, knockdown efficiency assessment by RT-qPCR (two-way ANOVA *p < 0.05, **p < 0.01). TNBC cells were transfected with optimized SMARTpool siRNAs for 72 h. GAPDH was used as internal reference. Right panel, Log2 FC of ASAP1 upon knockdown. The results were expressed as mean ± SEM of three independent experiments. **c** Volcano plot of DEGs in ASAP1-depleted BT549, Hs578T, and SUM149PT cells. The red and blue dots indicate down- and upregulated DEGs, respectively, with *p* value < 0.05 and absolute Log2 FC >1. **d** Venn diagram of down- and upregulated DEGs in BT549, Hs578T, and SUM149PT TNBC cell lines. Upper panel, downregulated DEGs; lower panel, upregulated DEGs. Common DEGs denote DEGs popping-up in at least two cell lines.

Amplification or deletion of a gene copy may affect the expression of genes located outside the amplified/deleted region itself via indirect mechanisms [22, 23]. The ASAP1 gene, located at chromosome 8q24.21, encodes an Arf GTPase-activating protein that induces hydrolysis of GTP bound to Arf proteins. ASAP1 has been reported to be involved in signal transduction, membrane trafficking, and cytoskeleton remodeling [24] and promote proliferative, invasive, and metastatic phenotypes of various cancer cells [18, 25]. Whether CNA-driven ASAP1 amplification and overexpression influence gene expression at a genome-wide level in cancer cells is not addressed. To this end, we performed TempO-Seq-based targeted whole genome RNA sequencing in the three TNBC cell lines BT549, Hs578T, and SUM149PT that harbor ASAP1 amplification and high expression (Fig. 3e). The cells were transfected with siRNA targeting ASAP1 (siASAP1) or non-targeting siCtrl in biological triplicate, respectively. The transcriptome TempO-Seq assays were run at a read depth of 6.8 M per sample (Supplementary Fig. 3a), achieving reproducibility with Pearsonʼs *r* values over 0.95 (Supplementary Fig. 3b). Principal components analysis of global changes in gene expression clustered various gene expression patterns across cell lines and transfections (Supplementary Fig. 3c). Using DESeq2 package in R, the Log2 normalized transcriptome profiles displayed the differential effects of ASAP1 depletion on gene expression in the TNBC cells (Fig. 5a). siRNA-mediated knockdown reduced >45% of mRNA expression of ASAP1 in the TNBC cells (Fig. 5b, left panel), warranting the effective knockdown of ASAP1 itself (Log2 FC −0.7 to −1.5) in the TempO-Seq transcriptome panels (Fig. 5b, right panel). Genes with twofold changes (absolute Log2 FC ≥1) in down- or up-regulation (*p* value < 0.05) were selected in BT549 (311 down/495 up), Hs578T (133 down/117 up), and SUM149PT (500 down/401 up) cells, respectively (Fig. 5c). Venn diagrams extracted differentially expressed genes (DEGs) that were significantly down- or upregulated by ASAP1 depletion in the TNBC cell lines (Fig. 5d; Supplementary Table 4). Consequently, 95 DEGs were downregulated, and 79 DEGs upregulated in ≥2/3 of the TNBC cell lines, in total 174 DEGs, which were considered as common DEGs that were susceptible to the depletion of the amplification-dependent ASAP1.

### ASAP1 regulates cytokine and apoptosis signaling components that are associated with TNBC prognosis

**Fig. 6:**
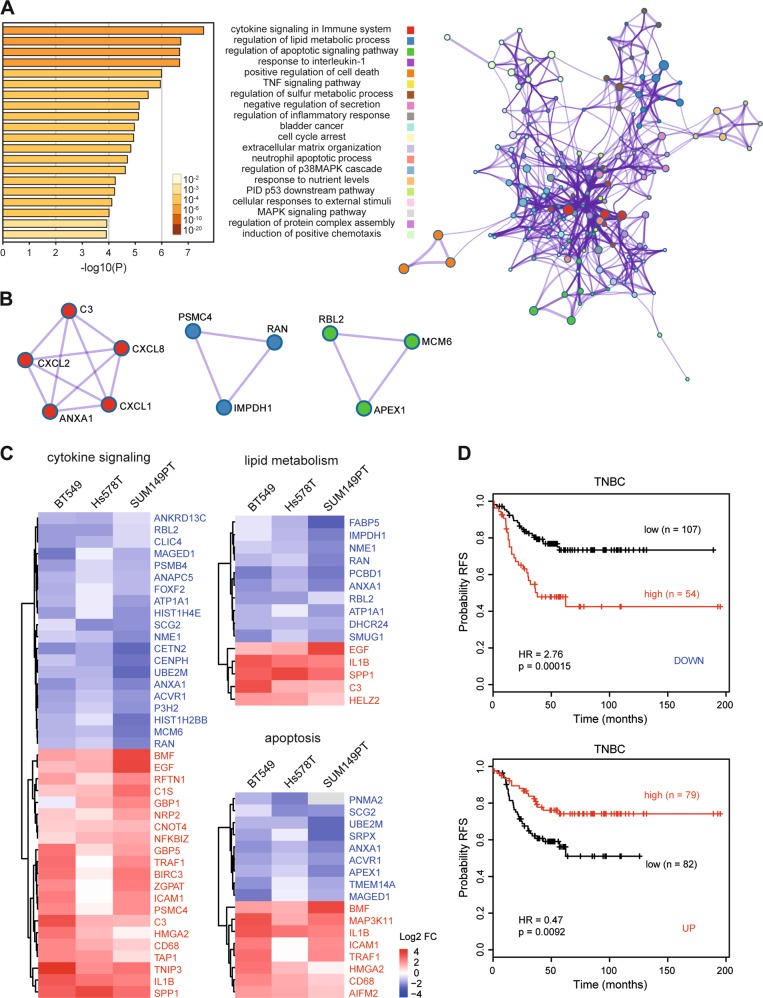
Metascape functional enrichment analysis and TNBC clinical relevance of ASAP1-regulated DEGs. **a** Top 20 clusters with their representative enriched term across input of 174 ASAP1-regulated DEGs. Left panel, heatmap of the 20 enriched terms. One term per cluster, colored by *p* values. Log10 (*p*) is the *p* value in log base 10. Right panel, network of the 20 enriched terms, colored by cluster ID, where nodes that share the same cluster ID are typically close to each other. **b** Representative Molecular Complex Detection (MCODE) network nodes, showing the ASAP1-regulated DEGs densely connected. **c** Log2 FC clustering of DEGs involved in cytokine signaling, lipid metabolism, and apoptosis pathways. Blue, negative regulators; red, positive regulators. **d** Relapse-free survival (RFS) KM curves of the DEGs from panel c in patients with TNBC. Mean expression of positive DEGs (DOWN) or negative DEGs (UP) was used.

Next, to evaluate the biological functions of 174 common DEGs that were susceptible to ASAP1 depletion, we conducted Metascape Pathway and process enrichment analysis integrating the gene ontology sources, including GO Biological Process, KEGG pathway, Reactome Gene Sets, Canonical Pathways, and CORUM [26]. Top 20 clusters were defined with their representative enriched terms (Fig. 6a, left; Supplementary Table 5), including cytokine signaling pathways (cytokine signaling in immune system, response to interleukin-1, TNF-signaling pathway, and regulation of inflammatory response), metabolic processes (regulation of lipid metabolic process and regulation of sulfur metabolic process), apoptosis signaling pathways (regulation of apoptosis signaling pathway and positive regulation of cell death), MAPK pathways (regulation of p38 MAPK cascade and MAPK signaling pathway), cell cycle arrest, and P53 downstream pathway. Furthermore, network enrichment captured the interactions between the 20 clusters, as visualized using Cytoscape (Fig. 6a, right). Strikingly, among the 20 clusters, the cytokine signaling in immune system, regulation of lipid metabolic process, and regulation of apoptotic signaling pathway were most significantly enriched. Protein–protein interaction clustering algorithm identified neighborhoods within the networks where the ASAP1-regulated genes were densely connected, such as ANXA1, C3, CXCL1, CXCL2, and CXCL8 node (involved in immune response), IMPDH1, PSMC4, and RAN node (involved in nucleotide and protein metabolism), and APEX1, MCM6, and RBL2 node (involved in cell cycle G1/S phase transition) (Fig. 6b). These results revealed the novel and essential biological functions of ASAP1 in multiple molecular pathways.

Around 45% of ASAP1-regulated genes (78/174) were involved in cytokine signaling pathways (Supplementary Table 6), being interactive in cytokine signaling in immune system, response to interleukin-1, TNF-signaling pathway, and regulation of inflammatory response. In regulation of apoptosis signaling and cell death pathways, 33 genes were modulated by ASAP1 depletion. Unsupervised hierarchical heatmap classified the 21 positive and 20 negative regulators in cytokine signaling pathways, 5 positive and 10 negative regulators in lipid metabolism pathway, and 8 positive and 9 negative regulators in apoptosis signaling pathways (Fig. 6c), in the BT549, Hs578T, and SUM149PT TNBC cell lines. As cytokine signaling pathways, e.g., TNF-signaling pathway, mediate extrinsic/intrinsic apoptosis [27], crosstalk might exist in the ASAP1-regulated cytokine and apoptosis signaling pathways. Indeed, among 33 ASAP1-regulated apoptosis genes, 23 genes (~70%) were interactive in cytokine signaling pathways (Supplementary Table 6), further stressing the implication of ASAP1 as a driver gene in cell survival and growth. Crosstalk was also observed between ASAP1-regulated genes in lipid metabolic process (17/31, ~50%) and cytokine signaling (Supplementary Table 6). To explore the association between these ASAP1-regulated cytokine, lipid metabolism and apoptosis genes and the survival of patients with TNBC, we performed Kaplan–Meier (KM) survival analysis using KM plotter [28]. The results exhibited that, based on the mean expression of the selected genes in TNBC tumors, a low expression level of the negative regulators, which were downregulated under ASAP1 depletion, predicted longer relapse-free survival of TNBC patients with statistical significance (logrank *p* = 0.00015, HR = 2.76) (Fig. 6d). In contrast, low expression of positive regulators, which were upregulated by ASAP1 depletion, implied significantly worse prognosis (logrank *p* = 0.0092, HR = 0.47).

Collectively, our data suggested that amplified ASAP1 is a key driver in TNBC progression by negatively regulating cell death pathways. The ASAP1-regulated genes in cytokine, lipid metabolism, and apoptosis signaling pathways were clinically relevant to TNBC relapse-free survival.

## Discussion

TNBC is characterized by its inter-tumoral heterogeneity. Based on gene expression signatures, TNBC has been further classified into more than six molecular subtypes [29], Yet, no effective molecular targeted therapies are currently available in the clinic for this type of BC. Although TNBC demonstrates substantial genetic alterations [30], only two genes, *TP53* and *PIK3CA*, have been found with mutation frequency in ~10% of TNBC patient tumors [6], indicating other driver mutations involved in TNBC progression. While amplification frequently occurs in cancer genomes, amplified genes are not always overexpressed [31]. A recent study has applied integrative analysis combining gene expression, miRNA and copy number variation genomic profiles for TNBC patients (*n* = 137) from TCGA to reclassify TNBC inter-tumoral heterogeneity [32]. As overexpression is a requisite for amplified genes to function as drivers in cancer, we attempted to identify TNBC candidate driver genes by integrating DNA copy number change and mRNA expression omics data across 222 TNBC cases (TCGA dataset *n* = 118 and Metabric *n* = 104). Using recurrent event calling algorithm ADMIRE analysis and transcript expression quantification, we consequently identified 138 genes with frequent focal CNGs and concurrent high expression in the cohort of TNBC tumors. Among these genes several are well-known oncogenes with involvement in BC, e.g., MYC, EGFR, CCNE1, and FGFR1, consistent to other studies [6, 15, 33]. We also identified many novel candidates, such as TMEM67, ANKH, BNIP1, CCT5, ASAP1, and IRF2BP2. KEGG pathways enrichment displayed the implication of the genes mainly in cancer-related or oncogenic signaling pathways. Our integrated genomic analysis reveals frequent CNGs and amplification as driver mutations in the TNBC genome. This large group of 138 candidate genes, displaying positive correlation in recurrent DNA CNGs and high RNA expression levels in the TNBC genome, may represent potential drivers during the course of TNBC progression.

CNAs often involve a number of genes within proximity in the recurrent regions of the same chromosome, resulting in gene co-amplification. Chromosomal region 8q24 amplification is common in many cancers. Several studies have reported CNG co-occurrence of numerous genes in this region, including MYC, ASAP1, PVT1, PTK2, BAI1, TRIB, and EXT1 [34–38]. Ehlers et al. reported that CNG in 8q24 strongly correlated with expression of ASAP1 but not that of MYC in uveal melanoma [34]. By contrast, other studies demonstrated that co-amplified MYC and PVT1 cooperatively promote tumor progression [37, 38]. In our TNBC cell lines, 13/20 cell lines represented co-amplification of MYC and ASAP1; however, our siRNA studies demonstrated that silencing of ASAP1 alone inhibited TNBC cell proliferation, independently of MYC. Although both MYC and ASAP1 displayed amplification-associated prognostic outcome in patients with BC, the expression of these two genes were poorly correlated in the same cohort of patients. Our results suggest that co-amplified MYC and ASAP1 genes are not necessarily coincidently expressed to promote TNBC tumorigenesis.

Our current siRNA-based proliferation screen validated several candidate genes promoting TNBC cell proliferative phenotype, including the known oncogenes MYC and EGFR and the novel candidates ASAP1, IRF2BP2, CCT5, and DRD1, indicating the implication of amplification-dependent drivers in TNBC proliferation. These genes (except for DRD1) acquired CNG/amplification and high expression in breast tumors. In particular, amplification of MYC and ASAP1 is highly frequent in TNBC (35% and 30.3%, respectively) compared with non-TNBC tumors (25.5% and 22.6%, respectively). The amplification-based MYC oncogene has been found in various solid tumors, including kidney and colorectal cancers and BC subtypes, including TNBC [39, 40], indicating MYC amplification as a frequent driver mutation in cancer. A recent study has revealed that MYC alterations are mutually exclusive with PIK3CA, PTEN, APC, or BRAF alterations [41], suggesting MYC amplification as a distinct driver mutation in the cancer genome. The novel candidate, ASAP1, has also been reported to be frequently amplified, accompanied by enhanced expression, in different types of cancers, including pancreatic ductal adenocarcinoma, prostate cancer, and melanoma [34, 42], exceptionally in primary BC where overexpression of ASAP1 was described to be independent of ASAP1 amplification [43]. These early results, though not reported for BC, underline the impact of ASAP1 amplification over the cancer genome. Here, our results initially interpret that, similar to other cancer types, the aggressive BC subtype TNBC acquires driver mutations, such as amplification-dependent overexpression of MYC and ASAP1, to progress, in spite of genetic heterogeneity. Of significance for ASAP1, we showed that knockdown of ASAP1 inhibits cell proliferation in different TNBC cell lines and high expression of ASAP1 is associated with poor MRFS of patients with TNBC but not ER^+^ BC, compelling the role of ASAP1 in TNBC progression, promoting not only proliferation but also metastasis.

In this study, we demonstrated for the first time the effect of ASAP1 on transcriptomic regulation in TNBC cells. ASAP1 (also named DDEF1 or AMAP1) was identified on the basis of Arf activity as a phospholipid-dependent Arf GTPase-activating protein and was found to bind to and be phosphorylated by Src family proteins and focal adhesion kinase and to associate with focal adhesions [44, 45]. ASAP1 has been shown to promote cell proliferation and invasion in different cancer cells, including lung, colorectal, prostate, and BC cells [18, 42, 46]. High ASAP1 expression level was found in and was required for invadopodia formation in invasive MDA-MB-231 TNBC BC cells, and interfering ASAP1-mediated protein complex inhibited metastasis of MDA-MB-231-derived xenografts [43]. Overexpression of ASAP1 has been reported to be associated with poor metastasis-free survival in prostate, colorectal cancer, and ovarian cancer and malignant phenotypes of primary BCs [18, 43, 47]. We showed that depletion of ASAP1 in different TNBC cells led to reprogramming of gene expression mainly in cytokine and apoptosis interactive signaling pathways, by upregulating positive regulators and downregulating negative regulators of the pathways. For instance, silencing ASAP1 downregulated the components in the identified mitotic cell cycle G1/S phase transition network node, including the antiapoptotic APEX1 that is abnormally expressed in numerous human solid tumors and positively correlated with cancer progression [48], the proliferation marker MCM6 that is predictive for poor prognosis in BC [49], and the direct AKT target RBL2 [50]. In addition, expression of genes involved in lipid metabolic process was also vulnerable to ASAP1 depletion, suggesting the novel role of ASAP1 in metabolism-related tumorigenesis, as lipid metabolism has been linked to cancer development by causing abnormal expression of various genes and dysregulating cytokines and signaling pathways [51]. Most importantly, numerous ASAP1-regulated genes, particularly those that were downregulated when ASAP1 was targeted, displayed significant relevance to relapse-free survival of TNBC patients, indicating ASAP1 as an upstream regulator in driving TNBC progression.

Deconvolution of genetic alterations in cancer genome, such as focal CNAs, has provided an excellent possibility to classify new cancer subtypes and identify novel therapeutic targets for naïve resistant cancers [8, 9, 32]. Our work elucidated extensive amplification-dependent gene expression alterations in TNBC, revealing ASAP1 as a potential TNBC driver functioning upstream of cytokine and apoptosis genes in promoting proliferation and survival. ASAP1 emerges as a potential diagnostic marker as well as therapeutic target for cancer, as ASAP1 has been found to be implicated in multiple oncogenic processes in various cancers [18, 42, 47]. Our results suggest that targeting the upstream regulator ASAP1 and its downstream target genes may provide actionable therapeutic strategies for overcoming the intractable TNBC disease, as well as other resistant cancer types overexpressing ASAP1.

## Materials and methods

### Cell culture

Human TNBC cell lines BT549, Hs578T, and SUM149PT were provided by Erasmus Medical Center (Rotterdam, the Netherlands) and cultured in RPMI-1640 medium supplemented with 10% fetal bovine serum, 25 U/ml penicillin and 25 µg/ml streptomycin in a humidified incubator at 37 °C with 5% CO_2_. All cell lines were authenticated by short tandem repeat profiling and subjected to mycoplasma test using the Mycosensor PCR kit (#302108, Stratagene).

### Selection of amplification-dependent candidate driver genes in TNBC genome by integrated AMDIRE copy number region analysis and transcript expression quantification

ADMIRE analysis [20] was performed to detect genomic regions with recurrent CNGs across 222 triple-negative tumors (*n* = 118 from TCGA; *n* = 104 from Metabric). Segmented copy number profiles for both TCGA [15] and the discovery set of Metabric [2] were obtained and used as input for the ADMIRE analysis. ADMIRE was configured to control its false discovery rate at 0.01. The recurrently altered copy number regions identified by ADMIRE were filtered in order to enrich regions most likely to harbor driver genes. Importantly, ADMIRE regions may be nested within larger regions, where the higher nesting levels correspond to more focal and more frequently altered regions. Our filtering only kept the regions detected at the highest nesting level. In addition, regions were kept only if they spanned at least one, but no more than 100 genes. Next, the genes contained in those regions were identified and selected as candidate driver genes, if their mRNA expression profile showed a positive correlation with their copy number. For this, the RNA-Seq data for TCGA analyzed using RSEM transcript quantification and the microarray expression data for Metabric were applied. The correlation was tested using Spearman’s correlation, where the Log-ratio copy number estimates were correlated with the expression values. Correction for multiple testing was performed by controlling the false discovery rate at 0.1. The ADMIRE analysis and the subsequent filtering steps were performed separately for TCGA and Metabric, after which the resulting gene lists were merged.

### siRNA-mediated loss-of-function screen

The primary screen was carried out by use of siGENOME Human SMARTpool siRNAs (GE Dharmacon, Lafayette, CO, USA) targeting 138 ADMIRE candidate driver genes. In the validation screen, SMARTpool siRNA and single siRNA_1, _2, _3, and _4 that comprise the SMARTpool mix were used to validate each candidate hit. Cells were seeded overnight in 96-well plate at an optimized density for BT549 (8000 cells/96-well), Hs578T (8000 cells/96-well), and SUM149PT (10,000 cells/96-well), and transfected with 50 nM siRNA by transfection reagent INTERFERin (Polyplus-Transfection SA, Illkirch-Graffenstaden, France) according to the manufacturer’s instructions. We used a pool of 720 kinase siRNAs at stock concentration of 1 µM in our laboratory as a negative control (siCtrl), this has no significant effect on expression of any single kinase genes; siRNA against KIF11 was used as positive control. The medium was refreshed 24 h post-transfection and TNBC cells were transfected for 2 days and proliferated for 4 days under indicated condition. SRB colorimetric assay was used as read-out for cell proliferation [52].

### Real-time RT-qPCR assay

Real-time RT-qPCR experiments were performed as previously described [53]. The primer sequences used were: forward *CAGCCGGCGCTTCCC*, reverse *ATCAGAAAACGACCGGGACC* (human ASAP1); forward *CTGGTAAAGTGGATATTGTTGCCAT*, reverse *TGGAATCATATTGGAACATGTAAACC* (human GAPDH). Relative mRNA levels after correction for GAPDH control mRNA were expressed using the 2^−ΔΔCT^ method.

### DNA copy number alteration and mRNA expression profiling of candidate genes in breast cancer cell lines and tumors

DNA copy number data of candidate genes in 20 TNBC cell lines were obtained from online resources [54]. Log2-based RNA expression profiles of candidate genes in 52 BC cell lines was retrieved from our own established RNA-Seq data. CNAs and mRNA expression of candidate hits in 2173 breast tumors were obtained from dataset “METABRIC, Nature 2012 & Nat Commun 2016” in cBioPortal (http://www.cbioportal.org/) [17] and the largest dataset with available DNA copy number (*n* = 2173) and mRNA expression (*n* = 1904) profiles. The 2173 breast tumor samples were filtrated by the immunohistochemistry status of ER, PR, and HER2, resulting in 320 triple-negative tumors.

### Human whole transcriptome analysis of ASAP1 silencing effect

TNBC cells were seeded into 96-well plate and transfected with optimal SMARTpool siRNA targeting ASAP1 (siASAP1), and siRNA control (siCtrl), as described above. The experiment was performed in biological triplicate. Seventy-two hours later, cells were washed with PBS and lysed in 80 μl 1× BioSpyder lysis buffer. Lysates were frozen at −80 °C and shipped to BioSpyder technologies on dry ice for human whole transcriptome targeted RNA sequencing TempO-Seq analysis. Expression data for 21,111 transcripts were generated (BioSpyder Technologies, Inc., Carlsbad, CA, United States). Normalization and differential expression analysis were performed using DESeq2 package. Specifically, each siASAP1 condition was paired with the corresponding control siCtrl and the counts for each sample were normalized using the DESeq2 estimateSizeFactors function. Differential expression of each treatment relative to its respective control was measured using the Wald test. With baseMean of counts <10 filtered, genes that were regulated by siASAP1 with significance (*p* value < 0.05 and absolute Log2 FC > 1) were considered significantly DEGs.

### Gene functional enrichment analysis of ASAP1-regulated genes

The DEGs, up- or downregulated by silencing ASAP1, were uploaded to Metascape [26] (http://metascape.org). Pathway and process enrichment analyses were carried out with ontology sources of KEGG pathway, GO Biological Processes, Reactome Gene Sets, Canonical Pathways, and CORUM. All genes in the genome were used as the enrichment background. Terms with *p* value < 0.01, a minimum count of 3, and an enrichment factor >1.5 were collected and grouped into clusters based on their membership similarities. To further capture the relationship between the terms of pathways and processes, network of enriched terms were visualized using Cytoscape, where each node represents an enriched term and is colored by its cluster ID. Furthermore, if network contains between 3 and 500 proteins, protein–protein interaction enrichment analysis was carried out with BioGrid, InWeb_IM, and OmniPath to identify densely connected network components, presented in Molecular Complex Detection network node.

### Assessment of clinical relevance of candidate genes to survival prognosis of breast cancer patients

The functionally validated candidate driver genes were further evaluated using KM analysis for their relation to OS of 1981 BC patients according to their gene expression [17]. Microarray DNA expression results from BrCa Gene-Expression Miner were used to classify prognostic association of ASAP1 expression levels with MRFS of 257 TNBC patients (*n* = 257) and ER^+^ BC patients (*n* = 2519) using “optimal” splitting criterion [16]. The ASAP1-regulated DEGs involved in cytokine, lipid metabolism, and apoptosis pathways were assessed for their relation to relapse-free survival of TNBC patients by KM plotter using “Auto select best cutoff” [28]. Mean expression of DEGs was used to assess their prognostic significance.

### Statistical analysis

Pearson correlation analysis was performed using GraphPad Prism 7. Statistical analysis of all experimental data was performed using two-way ANOVA (*p < 0.05, **p < 0.01, ***p < 0.001). Data were expressed as mean ± SEM of three independent experiments. Significance was set at *p* < 0.05. The hierarchical clustering in heatmap was performed using CRAN pheatmap package in RStudio (version 0.99.887).

## Supplementary information

Supplementary Table and Figure legends

Supplementary Table 1

Supplementary Table 2

Supplementary Table 3

Supplementary Table 4

Supplementary Table 5

Supplementary Table 6

Supplementary Figure 1

Supplementary Figure 2

Supplementary Figure 3

## Data Availability

The accession number for the TempO-Seq data generated in this study is GEO Accession GSE134803.
