# Supplementary material for: Integrative analysis of genomic amplification-dependent expression and loss-of-function screen identifies ASAP1 as a driver gene in triple-negative breast cancer progression
Source: Oncogene. 2020 Mar 31;39(20):4118–31. doi: 10.1038/s41388-020-1279-3 (PMC7220851; doi:10.1038/s41388-020-1279-3)
Supplement: Supplementary file 1 — Supplementary Table and Figure legends [file 41388_2020_1279_MOESM1_ESM.docx]

**Supplementary Information**

**Supplementary Table and Figure legends**

**Supplementary Tables S1-6 (xlsx):**

**Table S1.** ADMIRE candidate driver genes for TNBC. **Table S2.** Primary hits of siRNA-mediated loss-of-function screen. **Table S3.** BC cell line panel. **Table S4.** siASAP1 targeted DEGs in three TNBC cell lines. **Table S5.** Enrichment of ASAP1-regulated genes in 20 clusters. **Table S6.** ASAP1-regulated genes in cytokine signaling, lipid metabolic process and apoptosis signaling pathways.

**Supplementary Figures S1-3 (pdf):**

**Figure S1.** KEGG pathway enrichment analysis of 138 candidate driver genes. Orange line indicated where P-value = 0.05, whereas cyan dots indicated the number of mapped genes in each pathway. Pathways involved in cancer progression were highlighted in red. **Figure S2.** Association of CNA-driven candidate hits, including oncogene MYC and novel driver gene ASAP1, with OS of 2173 breast cancer patients. Kaplan Meier plot was generated in cBioPortal using dataset “METABRIC, Nature 2012 & Nat Commun 2016”. **Figure S3.** Quality control transcriptomic TemO-Seq analysis. **(A)** Sequencing library size distribution across treatments. **(B)** Sequencing reproducibility. Pearson correlation coefficient r was calculated among each replicate against triplicate mean per treatment. **(C)** PCA across treatments using Log2 read counts. Red circle, cell line clustering; blue circle, treatment clustering.
