# Supplementary figures and images for: Integrative analysis of genomic amplification-dependent expression and loss-of-function screen identifies ASAP1 as a driver gene in triple-negative breast cancer progression
Source: Oncogene. 2020 Mar 31;39(20):4118–31. doi: 10.1038/s41388-020-1279-3 (PMC7220851; doi:10.1038/s41388-020-1279-3)

**Fig.S1**

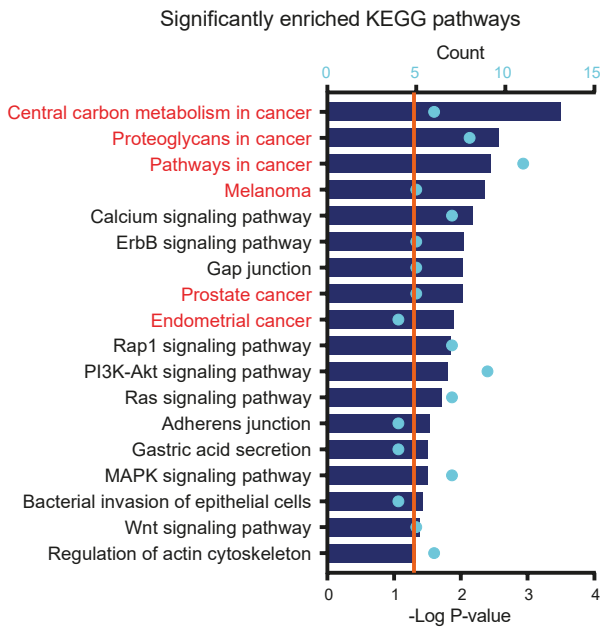

**He et al suppl. Figure S1**

Supplement: Supplementary file 8 — Supplementary Figure 1 [file 41388_2020_1279_MOESM8_ESM.pdf]

**Fig.S2**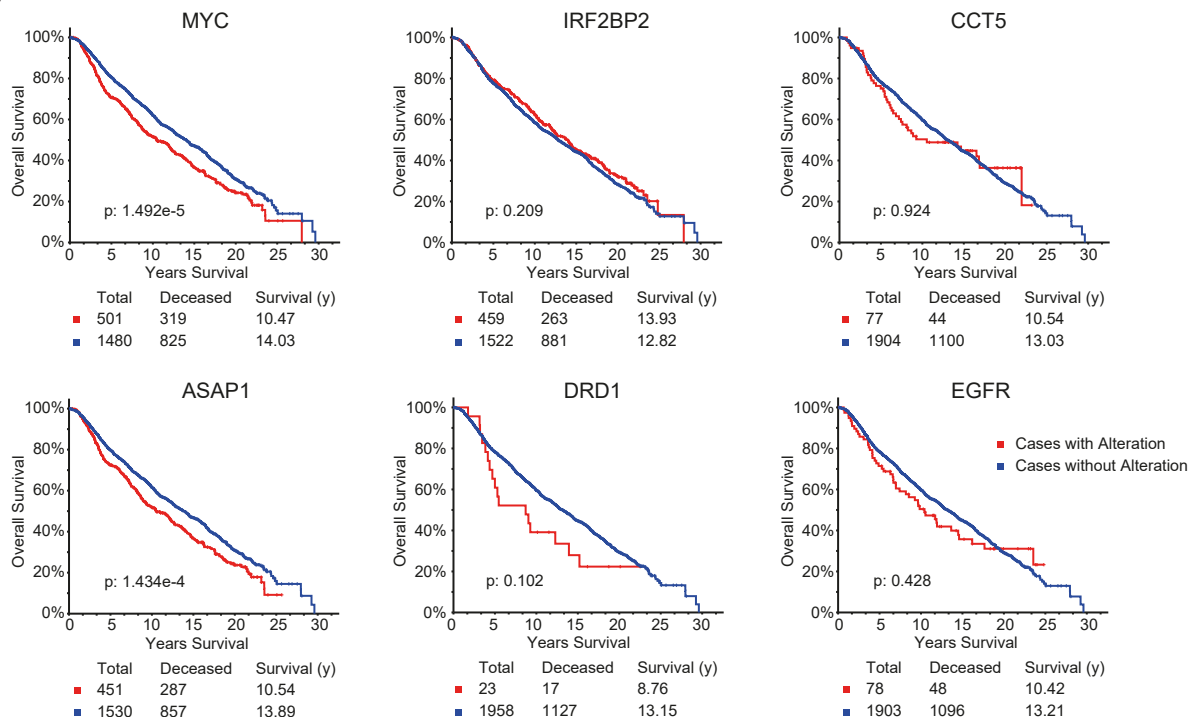**He et al suppl. Figure S2**

Supplement: Supplementary file 9 — Supplementary Figure 2 [file 41388_2020_1279_MOESM9_ESM.pdf]

**Fig.S3**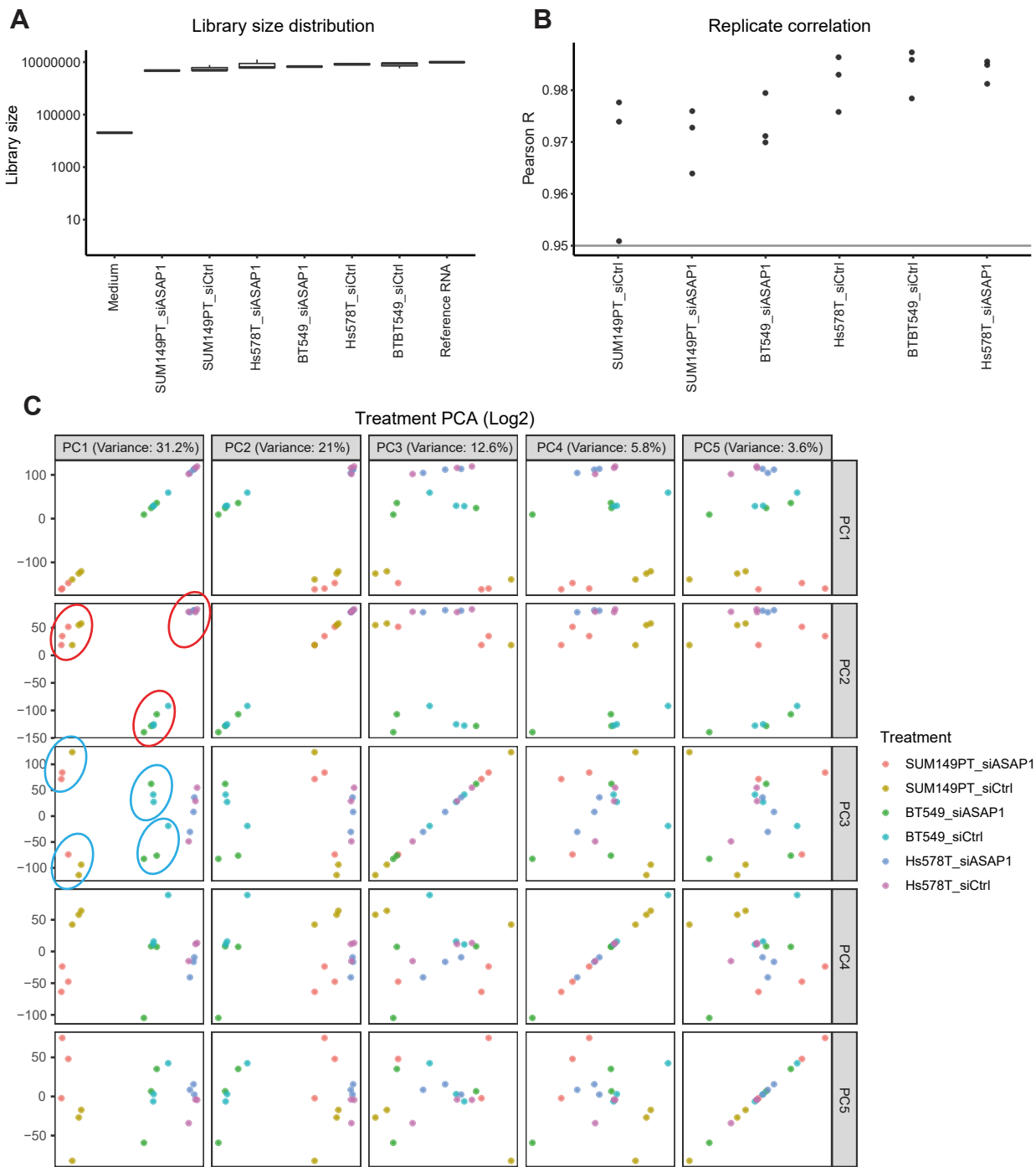**He et al suppl. Figure S3**

Supplement: Supplementary file 10 — Supplementary Figure 3 [file 41388_2020_1279_MOESM10_ESM.pdf]
